# Automated Chemical Sensing Unit Integration for Parallel Optical Interrogation

**DOI:** 10.3390/s19040878

**Published:** 2019-02-20

**Authors:** Ana L Hernandez, Fabian Dortu, Theo Veenstra, Paula Ciaurriz, Rafael Casquel, Iñaki Cornago, Hendrik V Horsten, Edurne Tellechea, María V Maigler, Fátima Fernández, Miguel Holgado

**Affiliations:** 1 Centre for Biomedical Technology, Optics, Photonics and Biophotonics Laboratory, Campus Montegancedo, Universidad Politécnica de Madrid, 28223 Madrid, Spain; rafael.casquel@upm.es; 2 Multitel, Parc Initialis 2, Rue Pierre et Marie Curie, 7000 Mons, Belgium; dortu@multitel.be (F.D.); vonhorsten@multitel.be (H.V.H.); 3 LioniX International BV, Hengelosestraat 500, 7521AN Enschede, The Netherlands; t.t.veenstra@lionix-int.com; 4 Naitec, Polígono Mocholí, Plaza Cein, 4, 31110 Noain, Spain; pciaurriz@naitec.es (P.C.); icornago@naitec.es (I.C.); eteiletxea@naitec.es (E.T.); fatima.fernandez.santos@gmail.com (F.F.); 5 Bio Optical Detection, Centro de empresas de la Universidad Politécnica de Madrid, Pozuelo de Alarcón, 28223 Madrid, Spain; mavi.maigler@upm.es

**Keywords:** optical sensor, sensing, resonant nanopillars, parallel detection, microfluidics, automatic system

## Abstract

We report the integration of an automated chemical optical sensing unit for the parallel interrogation of 12 BICELLs in a sensing chip. The work was accomplished under the European Project Enviguard (FP7-OCEAN-2013-614057) with the aim of demonstrating an optical nano-biosensing unit for the in-situ detection of various chemical pollutants simultaneously in oceanic waters. In this context, we designed an optical sensing chip based on resonant nanopillars (R-NPs) transducers organized in a layout of twelve biophotonic sensing cells (BICELLs). The sensing chip is interrogated in reflection with a 12-channels optical spectrometer equipped with an embedded computer-on-chip performing image processing for the simultaneous acquisition and analysis (resonant mode fitting) of the 12 spectra. A microfluidic chip and an automated flow control system composed of four pumps and a multi-path micro-valve makes it possible to drive different complex protocols. A rack was designed ad-hoc for the integration of all the modules. As a proof of concept, fluids of different refractive index (RI) were flowed in the system in order to measure the time response (sensogram) of the R-NPs under optical reflectance, and assess the sensors’ bulk sensitivity (285.9 ± 16.4 nm/RIU) and Limit of Detection (LoD) (2.95 × 10^−6^ RIUS). The real-time response under continuous flow of a sensor chip based on R-NP is showed for the first time, obtaining 12 sensograms simultaneously, featuring the unit as a potential excellent multiplexed detection system. These results indicate the high potential of the developed chemical sensing unit to be used for in-situ, multiplex and automatic optical biosensing.

## 1. Introduction

Detection systems based on biosensors have aroused notable interest during the last forty years, in particular due to the need to detect and monitor biochemical and biological compounds present in the surrounding environment, for example: man-made contaminants and biohazards in environmental systems; virus, bacteria and any kind of biomarker in human samples for clinical diagnosis and medical care; for quality control in the agricultural industry or even in the defense area for safety reasons [1,2]. 

Among the different types of biosensors, label-free optical biosensors can be highlighted [3], since they have been demonstrated to be efficient, with good sensitivities, and at a lower cost in comparison to other biosensors. In the recent years they have further evolved into easy-to-use, portable and manageable systems that can be produced at large scale [4,5,6]. These features have led them to be commercialized for the point of care testing (POCT) market [7], requiring systems with short time-to-result and on-site detection.

Some classical examples of label-free optical biosensors are based on Surface Plasmon Resonance (SPR) [8,9,10,11,12], Mach Zehnder interferometers [13,14,15,16,17], guided-mode resonance biosensor [18,19] photonic crystals [20,21,22,23,24], BICELLs [25], and ring resonators [26,27,28], that ultimately aim to be fabricated into integrated systems for POCT devices.

Here we present an optical sensing system combining microfluidics and optoelectronics system technologies (MFOS [29,30]) with the particularity of being used for the parallel interrogation as a function of time of multiple interferometers based on resonant nanopillars arrays (BICELLs) arranged on a quartz substrate [31]. This sensing system has been developed in the frame of the European Project Enviguard [32]. In former works we showed the effectiveness of optical interferometers based on R-NPs as chemical sensors [31,33] and as multiplexed label-free biosensors [34]. In this work, the R-NPs chips (sensing chip) are further integrated in a microfluidic system and coupled to an optical readout system. The microfluidic system is connected to four peristaltic pumps and a high precision multi-path micro-valve capable of driving different liquids to the sensing chip for managing complex fluidic protocols. The sensing chip is interrogated with an optical readout system capable of acquiring the 12 BICELL reflected spectra as a function of time and in parallel without requiring any complex light coupling that could limit the sensor throughput [35]. The detection is performed through a 2 × 12 optical fibers probe (12 fibers for illumination and 12 fibers for reflected light collection) connected to a 12 channels integral field spectrometer (1600 × 1200 pixels). The spectrometer collects the reflected spectrum of each of the twelve BICELLs interferometers in parallel with an optical bandwidth of 110 nm and with a wavelength resolution of 69 pm per pixel (110 nm/1600 pix).

This article describes the design of the sub-systems (sensing chip, fluidic system and optical readout system) and their integration into an automated opto-fluidic sensing unit with specifications (such as portability, easy-of-use, time response, sensitivity) suitable for the requirements of point-of-care or in-field testing applications. The figures-of-merit of the sensor are determined using salt solutions of controlled refractive indices (RI). We demonstrate an effective operation of the opto-fluidic sensor to be used in the future for target analytes monitoring. 

## 2. Materials and Methods

### 2.1. Chemicals

Sodium chloride (NaCl, >99%) was supplied by Sigma-Aldrich (St. Louis, MO, USA). Aqueous NaCl solutions were prepared dissolving different amounts of NaCl in ultrapure water in order to obtain different RI solutions.

### 2.2. Optical Transducer (Sensing Chip)

**Figure 1:**
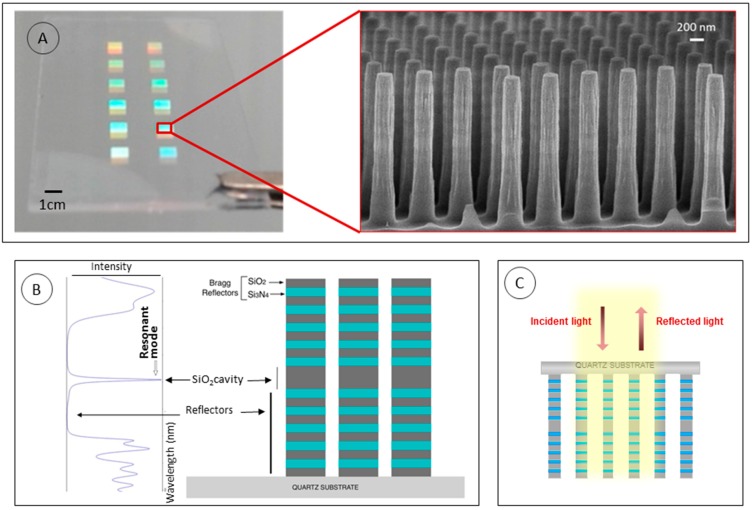
(**A**) Caption of the sensing chip with 12 BICELLs and SEM image of the R-NPs. (**B)** Example of the theoretical resonance mode formation when R-NPs are vertically interrogated, due to the central cavity between the Bragg Reflectors forming the R-NPs. (**C**) Vertical interrogation of reflected light from the back side of the chip.

The approach of the transducer for the proposed sensor relies on an optical interferometer which is based on biophotonic sensing cells (BICELLs) [25]. This is a novel concept of optical sensing surface composed by nanostructures distributed in independent arrays along the sensing chip, characterized vertically. In this specific case, the nanostructures proposed were resonant nanopillars over a quartz substrate which have been described in former works [31]. R-NPs are distributed into a quadratic lattice of 500 nm pitch (Figure 1A), and are composed of Bragg Reflectors pairs (BR) with two alternating dielectric materials (SiO_2_ and Si_3_N_4_) and a central cavity of SiO_2_. The reflectivity of the array of R-NPs is collected from the backside (Figure 1C). It is obtained the position of the resonant mode (due the central cavity), which depends on the refractive index of the surrounding media that can be tracked in order to obtain a refractive index sensor (Figure 1B). The same principle works for a biological recognition process.

For modeling the RN-Ps we developed theoretical calculations of the reflectivity of the arrays with the commercially available software RSoft [36]. The fabrication of the R-NPs arrays was detailed in the work of Cornago et al. [37], and is based on Laser Interference Lithography, which involves a single step of lithography for patterning relatively large areas, such as the chips used in this work. In that paper it is also showed the comparison of the R-NPs, vs. simple SiO_2_ nanopillars, justifying the application of the R-NPs in the project. 

In order to have a functional design, compatible with potential future multianalyte detection application, 12 BICELLs were included in the chip (for example, for measuring four analytes in triplicate). The dimension of the chip was 14 × 26 mm and BICELLs of 1 mm^2^ were distributed into two rows and six columns.

### 2.3. Bulk Sensing Experiment

**Figure 2:**
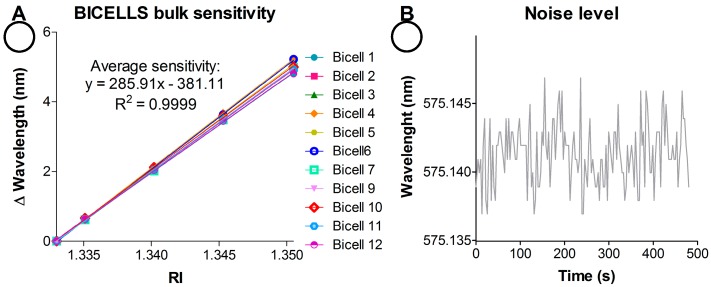
(**A**) BICELLs bulk sensitivity (Δnm vs. RI, BICELL 8 is omitted). (**B**) Detail of signal fluctuation of one BICELL.

For bulk sensing experiments, the wavelength shift of the resonant mode (Δnm) was monitored as a function of the RI of the liquid loaded in the system. For this purpose, the response as function of time (nm vs. time, sensogram) of each BICELL was simultaneously recorded by flowing NaCl aqueous solutions of different RI (0–15% NaCl, 1.3330–1.3695 RI). Fluids were pumped sequentially, 10 min each, at 37 µL/min. Solutions were allocated in the different reservoirs of the automatic fluidic system. These sensograms are included in Figure 2.

For bulk sensitivity assessment, linear regressions curves were constructed by representing the wavelength shift (Δnm) vs. RI (RIUs, Refractive Index Units). The slope of the curves (wavelength shift per unit of RI, nm/RIU) represents the sensitivity (m) of the system for each BICELL. The sensor LoD was defined as the expanded uncertainty (U) of the resonant mode position (considering a coverage factor of 3 divided by the sensitivity (Equation (1)). The U was calculated as described in Equations (2) and (3) according to Ref. [38]:

(1) $$\mathrm{LoD}=\frac{U}{m}$$

(2) U=3u

(3) $$u^{2}=\frac{R^{2}}{12}+\frac{s^{2}}{n}$$

*S*^2^ was assessed from sensogram when a solution is flowing (Figure 2B) considering the signal fluctuation over 200 s (i.e., *n* = 66 data points).

*R* is the resolution of the system used to perform the measurements (i.e., spectrophotometer resolution).

To evaluate the mixing capacity of the automatic fluidic system (sample loop, see Section 3.1 and Section 3.5), solutions of 0 and 20% NaCl were mixed (50% each) prior being exposed to sensing chip (10 min, final concentration 10% NaCl). Subsequently, a 10% NaCl solution was pumped (10 min) in order to compare signal change of both solutions. Deionized water was flowed between the different NaCl solutions to obtain the baseline.

## 3. Results

Each specific element of the sensor module was independently developed; however, the measurements and specific features composing the final system were developed based on a central design agreed upon all the members participating in this work. 

### 3.1. Fluidic System

The fluidic design includes several reservoirs and pumping modes in order to be compatible with future applications. 

**Figure 3:**
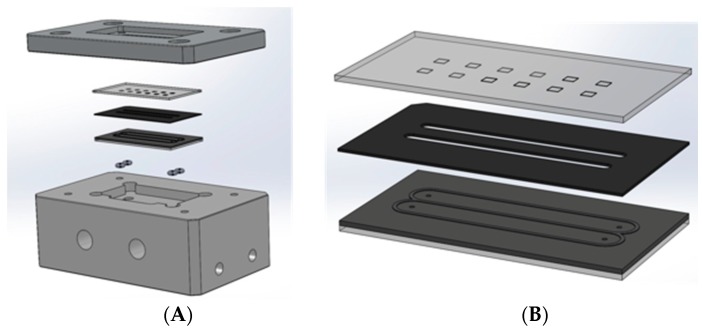
Design of the flow-cell of the fluidic system. (**A**) An exploded view, showing (from top to bottom) the chipholders lid, the sensing chip (BICELLs facing down), a gasket defining the flow-cell, the fluidic chip, O-rings for the seal between chip and the chipholder body, providing standard connections to the outside world. (**B**) Close-up from the three main parts defining the actual flow-cell: Sensing chip, gasket and fluidic chip (from top to bottom).

**Figure 4:**
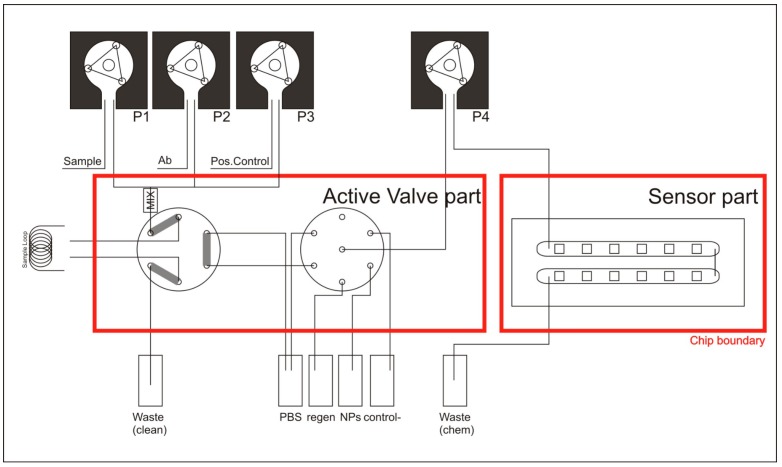
Schematic of the layout of the realized fluidic system. The three pumps upper left, are used to fill the sample loop with a mixture of solutions. The fourth pump is used to feed a selection of reagents through the sensing chip with the 12 BICELLs. The selection of the reagents is done through a selection valve (connected to containers), which also can connect to the sample loop to take up actual sample (There are in total seven reservoirs although in the scheme only six are represented).

The fluidic system consists of two main parts; the flow-cell containing the sensing chip (Figure 3) and secondly the liquid control with which the immunoassay protocol may be implemented (Figure 4). 

The flow-cell was realized by compressing a fluidic chip (with in- and outlets) onto the sensing chip with a gasket in between. Cutouts in the gasket define the channel of the flow-cell (Figure 3B). To ensure a leaktight seal between these three parts (sensing chip, gasket and fluidic chip), they are compressed tightly, in between the so-called chipholder body and the chipholder-lid (Figure 3A). The lid has a cutout, providing optical access to the BICELLs, whereas the chipholder body is equipped with standard fluidic ports (1/4-28) to accommodate fluidic access to the flow-cell.

Therefore, all BICELLs can be interrogated simultaneously, providing a potential multiplexing capacity if different analyte were measured in each BICELLs.

The second part of the fluidic system is the liquid control. In a predefined timing and order, a series of liquids (reagents as well as sample) can be sent through the flow-cell. Through this sequence, it could be performed a fluidic protocol specifically designed for the convenient application.

This chemical sensing fluidic system could be used as a biosensor for biochemical and biological detection. Therefore, the fluidic system includes seven reservoirs in order to automatically run different sequences: i.e., running buffer, sample, regeneration and other additional solutions for immunoassay applications such as positive or negative controls, stabilizing solutions, etc. 

The fluidic system includes a procedure to mix two solutions (sample loop), prior to be exposed to the sensor surface, if so requires the designed protocol.

The scheme in Figure 4 shows the functional design of the liquid control system. Pumps P1, P2 and P3 are used to fill a sample loop with a mixture of solutions (P2). Sample loop can also be filled with an additional solution (P3). The two liquids are thoroughly mixed before they enter the sample loop by using a micro-mixer. Any excess liquid volume is transported towards a waste-container.

The red lines indicate the boundaries of which functionality is in the two fluidics parts (liquid control and flow-cell). Once the sample loop is filled, pump P4 is used to direct reagents and sample-loop from their respective containers to the flow-cell. The sequence and timing of the reagents-flow leads to the execution of a potential designed specific application. Liquids that passed the flow-cell are collected in a waste container.

#### The Rotary Valve

The functionality of the fluidic system shown in Figure 4 stems largely from the mixer and the sample-loop valve, and the selection valve. In order to reduce the overall system size as well as to reduce the internal (dead) volume, the functions of these three components were combined in a single newly developed rotary valve. 

The rotary valve consists of a glass chip and a rotor, which are pressed tightly on to each other by means of a chip-holder. Channels in the surface of the rotor connect different ports on the fluidic chip at different orientations. 

**Figure 5:**
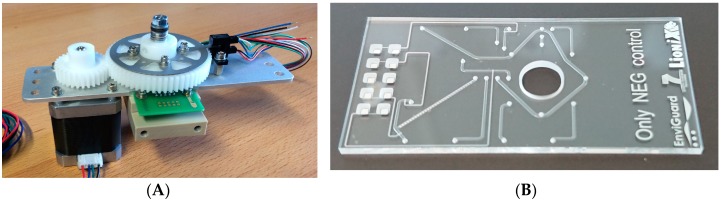
(**A**) Photograph of the assembled rotary valve. The rotor is turned by means of the stepper motor, which drives the large gear on top of the rotor. A light switch is used to detect the zero-position of the valve. Disc-springs are present on the axle to provide the compression force needed between the rotor and the valve-chip in order to obtain a leaktight connection. (**B**) Valve fluidic chip.

The design of the fluidic chip and the accompanying rotor (Figure 5) was customized to an immunoassay potential protocol that is to be automatically executed. The steps of an implemented protocol example are summarized in Table 1. For detailed explanations of the otatory valve design, fabrication and functioning, see Appendix A of this article.

### 3.2. Optical Readout System

**Figure 6:**
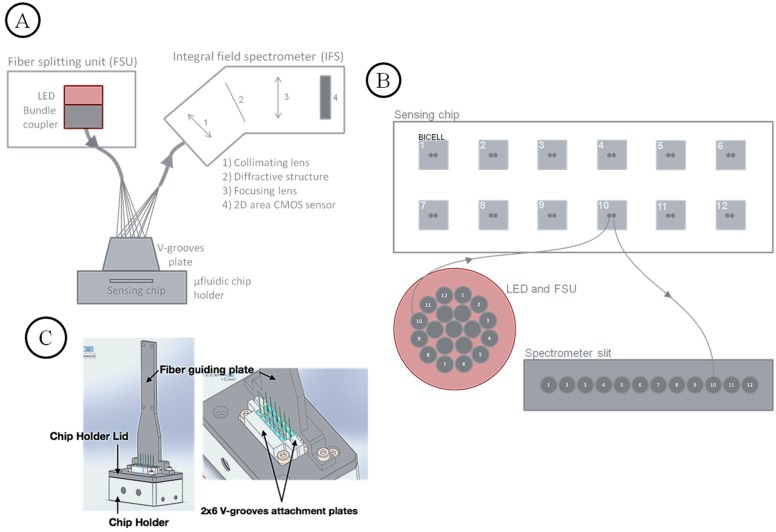
(**A**) Overview of the optical readout system. (**B**) Fiber layout transformation: LED (circular layout), Sensing chip (rectangular layout), Spectrometer entrance slit (linear layout), as explained in the above text. (**C**) Close-up on the V-groove plate holding the illumination and collection fibers close the sensing chip.

The optical readout system is built around an optical fiber bundle probe composed of 2 × 12 multimode fibers optically coupled in a reversible way to the sensing chip for BICELLs illumination and reflected light collection. The twelve illumination fibers of the probe are coupled to a broad band light emitting diode (LED) in the fiber splitting unit (FSU). The twelve collection fibers are coupled to a 12-channels integral field spectrometer (IFS) (Figure 6A). Illumination and collection fibers are grouped in pair, with each pair facing a BICELL (Figure 6B). 

The optical probe is a V-groove plate assembly which can be seen as an interface between the 2 × 6 bi-dimensional array layout of the sensing chip, and the fiber layouts of the FSU (circular layout) and the IFS (linear layout). The FSU is composed of a 19 fibers circular bundle directly glued on the LED surface of which only 12 fibers are used for illumination, while the entrance slit of the IFS is composed of a 1 × 12 one dimensional fiber array.

The 12-fibers V-grooves ensure the good lateral and vertical alignment of the fibers with respect to the BICELLs chip. The distance between the tip of the fibers and the BICELL surface has been optimized in order to maximize the collected signal (light reflected by the R-NPs). The fragile fibers are protected by two fiber guiding plates that are directly attached to the chip holder lid described in Figure 3. The light reflected by the BICELLs is captured by the collection fibers and directed to the IFS for spectral analysis. The IFS is composed of collimating/focalizing lenses, a diffraction grating and a 2D CMOS sensor (covers 110 nm of spectral range, from 525 to 635 nm). The IFS makes an image of the 12 fiber tips (slit) through a transmission grating to produce 12 parallel dispersion spectra on the CMOS sensor. It is capable to acquire 12 spectra in parallel covering a spectral range from 525 to 635 nm overlapping the resonance bandwidth of the R-NPs. The spectral resolution is 69 pm/pix (110 nm/1600 pix). 

**Figure 7:**
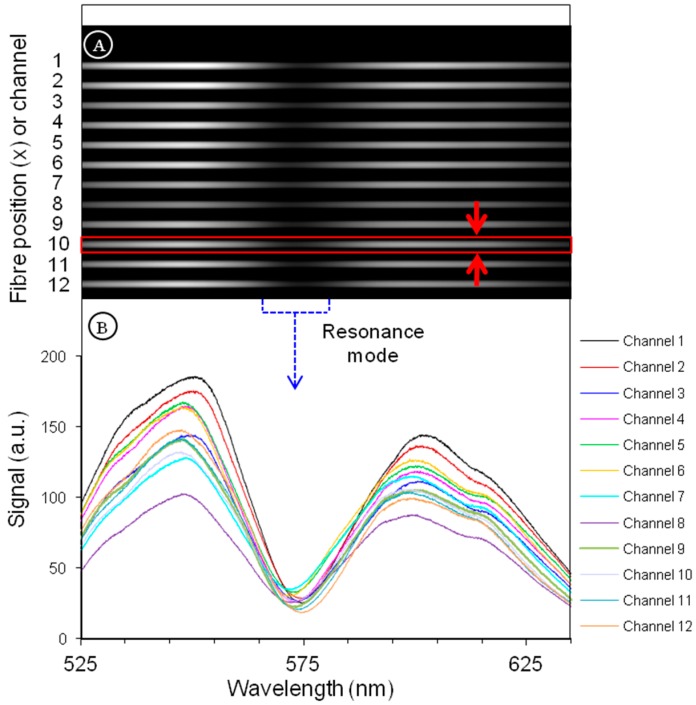
(**A**) Full field image captured by the camera. Each horizontal gray track corresponds to a spectrum of a BICELL. The shadow area in the middle of the image corresponds to the position of the resonance mode (it is a dip because light is transmitted through the BICELL at resonance). The height of the red rectangle around channel 10 illustrates the pixel length (~50 pixels) over which the signal is integrated to obtain the one dimensional spectra. (**B**) Extracted one dimensional spectra from the image. Each dip is fitted with a Lorentzian (not shown) in order to extract the resonance position with a high precision.

The images captured by the CMOS sensor are automatically processed in a computer-on-chip (ARM CPU). The spectra are extracted (Figure 7B) from the full field image (Figure 7A) by integrating the signal of each channel over ~50 pixels along the *x* axis. The resonance positions of the 12 spectra are extracted with high precision by fitting Lorentzian curves using a Levenberg-Marquardt fitting algorithm. The fitting gives a precision on the resonance position more than 10 times better (≤1 pm) than the CMOS sensor limited pixel resolution (69 pm). The determination of the sensor sensitivity is detailed in Section 3.4. The acquisition rate, including dip fitting, is ~2 spectra set per second (=24 spectra/s).

### 3.3. Housing and Integration of the Parts (Exchange Chip)

We designed and developed a proper housing for the integration of the different parts of the opto-fluidic sensing system. This allowed to accommodate all the components and to fulfill some requirements such as easy access to the replaceable parts or stability for the transportation and measurements in the field.

**Figure 8:**
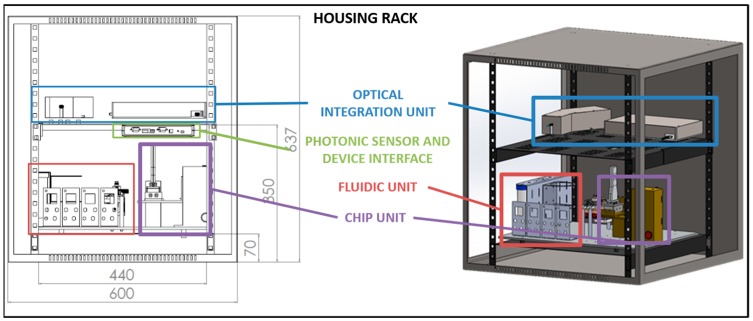
CAD design for the distribution of the different units within the system in several shelves of a commercial rack.

**Figure 9:**
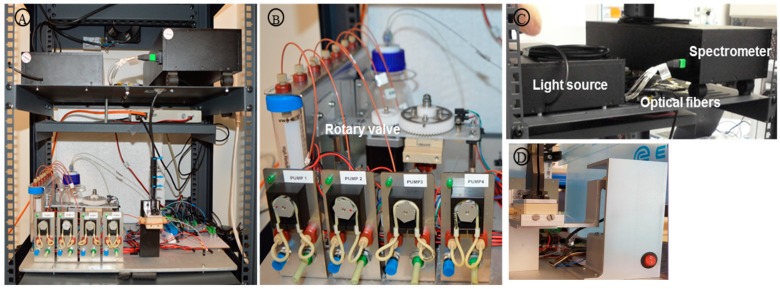
(**A**) Optofluidic sensing system integration. (**B**) Fluidic unit: rotary valve, peristaltic pumps and reservoirs. (**C**) Optical Integration Unit. (**D**) Chip and exchanging mechanism unit.

For this purpose, it was used a commercial rack, which has several advantages: standard sizes, multitude of vendors and qualities as well as different customization options (door positions, wire access or accessories) (Figure 8 and Figure 9). The different shelves included in the rack were fixed to its profile to allow to integrate each system in a different shelf and to modify the distance between them according to the requirements of the design. For this integration, a 19 inches rack (600 × 500 × 504 mm—9U) was used, fabricated in steel sheet (1.5 mm thick), and with removable lateral and rear panels to reach and change the removable components (reservoirs and sensing chip) easily. The selected rack contains three different shelves: two shelves of 2U (445 × 470 mm) to support the fluidic system unit and sensing chip unit and the optical readout unit and one shelf of 1U (445 × 270 mm) to hold the photonic sensor and device interface. The trays were mechanized to fix the elements for the optical system and to guide the fibers between levels, as well as to accommodate an aluminum plate that supports the microfluidic system. 

Figures below show the CAD design for the distribution of the different units inside its housing (Figure 8) and the results after its installation (Figure 9).

On the other hand, the chip exchanging mechanism was designed in order to facilitate the operator to access the chip without compromising the fiber optic movements and with no need to sustain the microfluidic holder, so a shorter exchange chip time is expected. The solution developed was an automatic system based on a stepper motor and two optical switches, along with an aluminum structure that maximizes the effective stroke to allow the chip holder to reach its lowest position (Figure 9D). 

**Figure 10:**
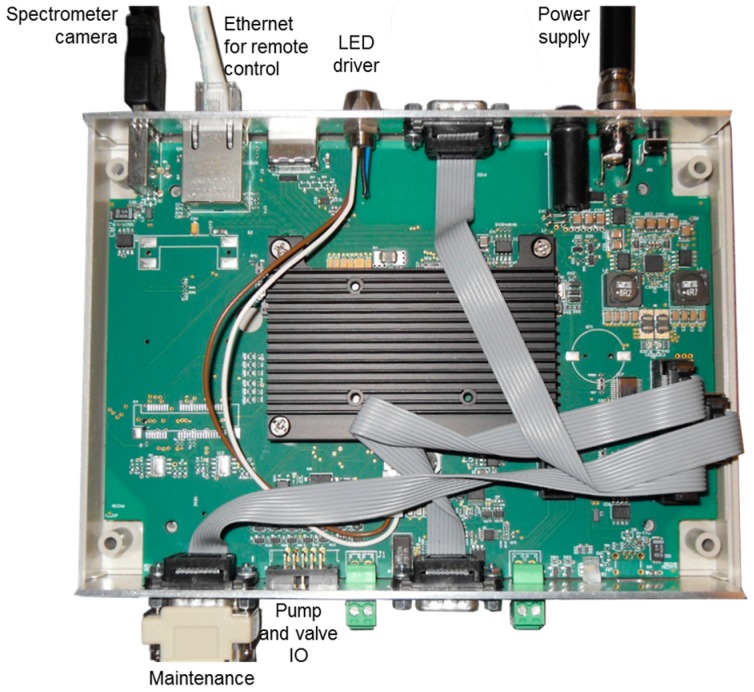
The main controller mother board.

The whole system is driven by a computer-on-chip (ARM CPU) mounted on a specifically designed motherboard with eight General Purpose Input Output controls (GPIO), plus a LED driver and serial and Ethernet (TCP/IP) communication bus. The GPIOs are used to control the fluidic pumps and valve with user configurable ON/OFF time sequences for implementing a bioassay protocol. The LED driver delivers the necessary current to the LED for BICELL illumination. Two communication protocols, Modbus and SCPI (IEEE 488.2), are implemented as a server over TCP/IP so that the whole system can be controlled by an external client. (Figure 10).

### 3.4. Bulk Sensing Experiments

**Figure 11:**
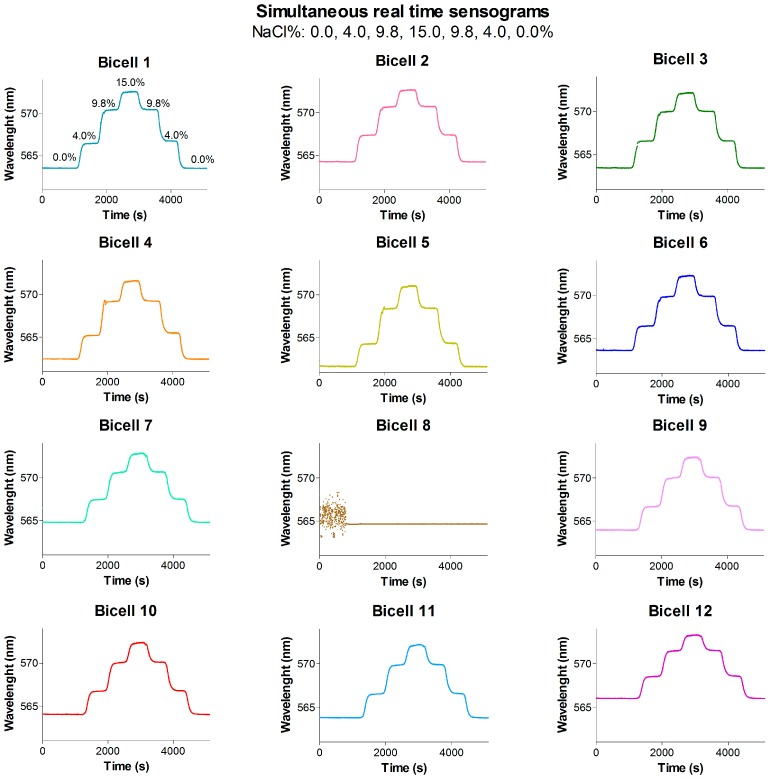
Sensograms (mn vs. time) of the 12 BICELLs acquired simultaneously under continuous flow conditions. Response to RI 1.3330, 1.3399, 1.3486 and 1.3565 (0.0–15.0% NaCl *w*/*v*). (BICELL 8 gives no signal due to the breakage of its illuminating optical fiber).

In order to assess the overall performance of the integrated system as well as sensor sensitivity, a bulk sensing experiment was performed. For it, several RI solutions (1.3330–1.3565) were automatically loaded in the flow-cell from the different containers. Changes in the resonance mode position (*λ*, nm) were monitored (nm vs. time, sensogram). As presented in Figure 11, the developed optical readout system allows recording the 12 BICELLs signal simultaneously, featuring the unit as an excellent potential multiplexed detection system. 

Moreover, the reflectivity time response of the R-NPs at normal incidence is showed for the first time. As observed in our former works, for higher RI, resonance position shifts to higher wavelengths. It was observed a total increase of 8.1 ± 0.5 nm from the solution with the lowest RI to the highest RI solution. A decrease in linearity was found for high RI (i.e., 1.3565), so lower RI changes were assayed subsequently (1.3330–1.3505, 0–10% *w*/*w* NaCl). 

The sensitivities of all BICELLs are represented in Figure 2A. An average sensitivity of 285.9 ± 8.8 nm/RIU was obtained for this sensing chip with excellent linear correlation (*R*^2^ = 0.9999). Besides, low standard deviations (~0.0023 nm) were registered for these measurements (Figure 2B). These results reveal the linear and very stable real time response of the sensor. Considering Equations (1)–(3), average calculated LoD values for this chip resulted in 2.95 × 10^−6^ RIUs. In this case the uncertainty regarding the resolution (*R*) of the readout system (1 pm) was neglected due to its low value in comparison to *s*^2^ and therefore *R* had a very low weight in the Equation (3). Furthermore, these experiments were carried out with a considerable number of sensing chips (*n* = 20). Table 2 shows the reproducibility of resonance position, sensitivity and LoD among the BICELLs of the same chip (intra-chip) as well as among different chips (inter-chip). 

### 3.5. Evaluation Mixing Process

Finally, the micro-mixer was evaluated. 20% and 0% NaCl solutions were thoroughly mixed before they enter the sample loop by using the micro-mixer in order to obtain a 10% of NaCl solution. The mixture was compared with a solution of 10% NaCl. 

**Figure 12:**
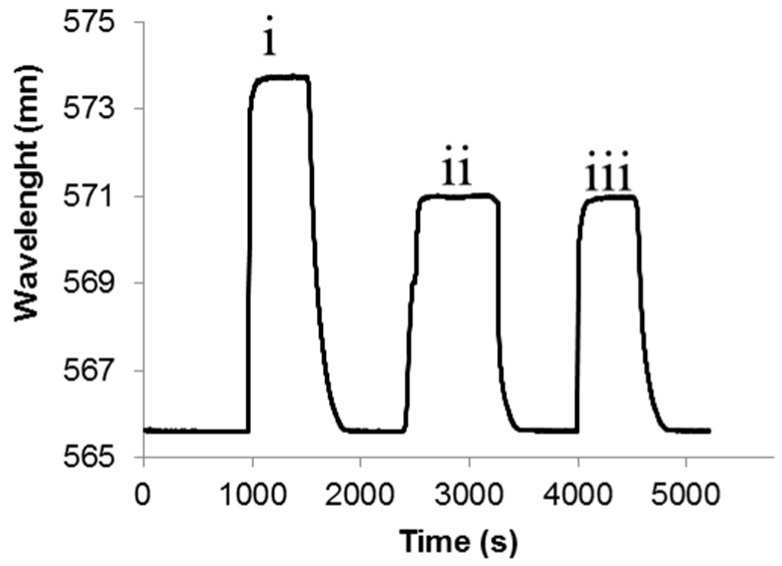
Sensogram (nm vs. time) of BICELL 1: Comparison of signal provided by (i) a 20% NaCl solution; (ii) a mixture of 20% NaCl and 0% NaCl in the micro-mixer (i.e., 10% NaCl solution); and (iii) a 10% NaCl solution.

As shown in Figure 12, the variation of *λ* (nm) obtained by the mixture and the 10% NaCl control of solution was the same in each BICELL, proving the proper working of the mixer and the homogeneity of the mixture.

## 4. Discussions

The presented results demonstrate that the sensing unit for parallel measurements as a function of time has been successfully developed. Sensing chip, fluidic system and optical readout system have been integrated in one single rack platform under sensing specific aims. 

We have demonstrated to achieve a LoD of 2.95 × 10^−6^ RIUs, which can be improved by means of optimization of R-NPs performance design (diameter, height, and lattice parameter) as well as modifying parameters of the spectrometer from the IFS (resolution, integration time). 

The low % CV values obtained for all the parameters (Table 2) highlight the reproducibility of the sensor as well as the transducer fabrication process and the homogeneity among the BICELLs. Average values for sensitivity inter-chip (299.2 ± 10.5 nm/RIU) and LoD (3.32 × 10^−6^) are in the range of other reported optical sensors based on periodic arrays, such as photonic crystals and ring resonators (10^2^–10^3^ nm/RIU and 10^−4^–10^−5^ RIUs respectively) [39,40,41].

Nevertheless, in order to be able to draw reliable information from the sensor performance other variables that influence the calculation of the figures of merit (Equations (1) and (3)) must be considered. For example, the number of measurements during a certain time, (66 measurements and 200 s in this work) for each fluidic solution. By increasing the number of measurements, the uncertainties can experience a considerable decrease (considering a constant *s*^2^ along the time), leveraging thus the LoD. 

However, by taking an unmet number of measurements, on one hand the use of the sensor could become extremely time-consuming, negating its regular use and on the other hand the stability of the measurements would not be guaranteed. We have verified the stability of each fluid for 200 s. 

In order to increase the number of measurements during those 200 s and therefore decrease the uncertainty, one solution could be to decrease the integration time of the readout system for each measurement. For that it would be necessary to decrease the number of the optical channels. To multiply by 3 the integration time it would be necessary to reduce the 12 existing channels (for the 12 BICELLs) to four. However, this would limit the potential multiplexing capacity of the sensor. 

The calculation of the performance of a sensors must be a commitment among different parameters (time, multiplexing, stability, limit of detection, etc.) to be considered for the different purposes for which the sensor is developed.

The output of this work is the integration of the fluidic system and the optical read out system of high resolution that enables the development of a sensing unit that can be potentially applied, to different fields of interest, especially for biological sensing, by adapting the specific needs.

## 5. Conclusions

In this paper we report the integration of the different elements composing a unique chemical sensing unit for parallel detection revealing a good bulk sensing performance, which could be used for multiplexed analyte detection.

In addition it must be recalled the potential of the described work, since the different modules design and integration can be particularized for multiple application in a wide range of fields. (Different R-NPs materials, dimensions, the number of BICELLs and optical channels, resolution and integration time of the optical readout system, are some of the parameters that could be modified according to the different specific areas of application).

Therefore, the demonstrated system can be used for different applications, thus enhancing its potential impact. In fact, the parallel detection of different biomolecules could be of paramount importance to further purposes (medical, water toxicity, air pollutants, agricultural quality assessment, industrial process control). Moreover this rack can be transported to different environments to provide the final users with an in-situ detection system.

Finally, it should be highlighted that the independently developed technologies here can also be applied to further commercial systems where such an integrated module is necessary.

## Figures and Tables

**Table 1:** The potential protocol to which the rotary valve was designed for different fluids measurements (an example is described in Table 1).

Step in Protocol	Function
01	PBS to sensing chip, (Sample loop is open for loading in this position)
02	Sample or additional solution 1 from sample loop
02a	Additional solution 2
03	PBS
04	Additional solution 3
05	PBS
06	Regeneration buffer
07	PBS

**Table 2:** Reproducibility of BICELLs response within one chip (intra-chip, number of samples = 11 BICELLs) and among different chips (inter-chips, number of samples = 11 BICELLs × 20 sensor chips = 220). LoD or resolution was calculated according to Equation (1) where sample size *n* = 66 in all cases.

	*λ* (nm)		Sensitivity (nm/RIU)			s_ave_(nm)	LoD (RIUs)	
	X ± S	%CV	X ± S	*R* ^2^	%CV	X ± S	X	%CV
Intra-chip	575.4 ± 0.9	0.15%	285.9 ± 8.8	0.9999	3.09%	0.0023 ± 0.0003	2.95 × 10^−6^	15.40%
Inter-chip	573.2 ± 2.7	0.47%	299.2 ± 10.5	0.9994	3.52%	0.0027 ± 0.0007	3.32 × 10^−6^	25.98%

**Figure A1:**
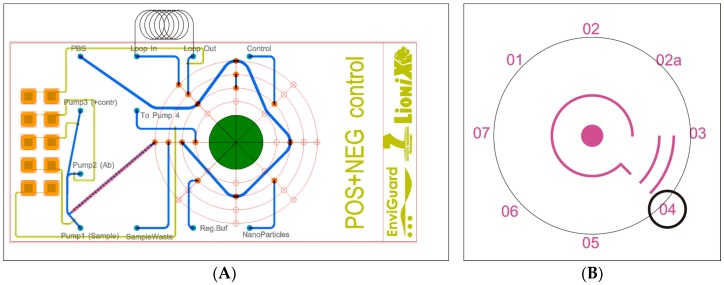
Design of the fluidic valve-chip (**A**) and the rotor (**B**). (**A**) Etched channels are in blue; connections towards the rotor are orange dots; connections towards the system are in light blue. The green circle is the cutout through which the rotor’s axe will pass. The mixing structure is indicated with the dotted line. (**B**) The channels on the rotor. The rotor is currently shown as in position “4” of the protocol.

**Figure A2:**
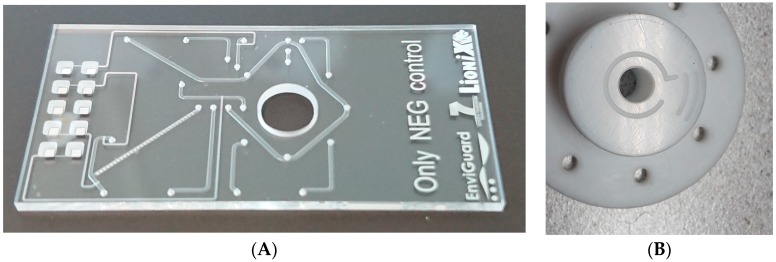
(**A**) Photograph of the valve’s fluidic chip. (**B**) Rotor.

**Figure A3:**
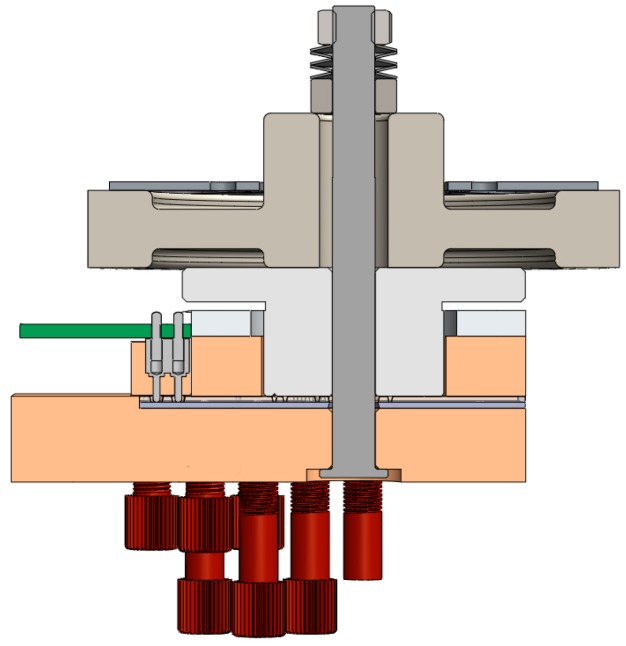
Cross-sectional view of the rotary valve. The fluidic connections are at the bottom side of the chip-holder. The central pin of the valve is fixed in the chip-holder and extends through the chip, the rotor and the gear. Spring discs are used to supply an even pressure on the rotor.

## Appendix A

For the design of the rotary valve only the sequence of a potential protocol is of interest. Also, the design has been done such that the valve always moves “forward”, preventing possible cross-contamination issues. This results in a design in which the main solution is connected to the outlet in multiple steps (Figure A1).

The valve-chip is fabricated through micromachining-techniques as HF-etching, powder blasting and fusion bonding, whereas the rotor is fabricated using standard machining techniques. To ensure a leaktight connection between the chip and the rotor, the rotor has been polished using down to one micron grit abrasives. The realized parts are shown in Figure A2.

With the realized chip and rotor, all necessary connections for a full execution of a selected fluidic protocol are made when turning the rotor in 8 steps of 45°. Details on the composed flow paths for each protocol step are described in Table A1. For the execution of a full measurement using a protocol, either step 02 or 02a is used. Position 02 corresponds of the situation where the contents of the sample-loop are transported to the sensor, where in position 02a the source is the container with an additional solution.

**Table A1:** Flow paths in the fluidic chip for each position of the multivalve.

Protocol Step	Flowpath through Fluidic Chip	Rotor Position
01		
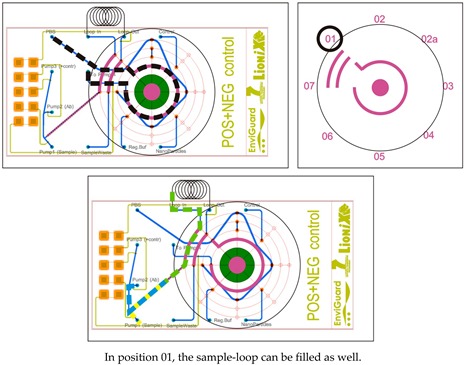
02		
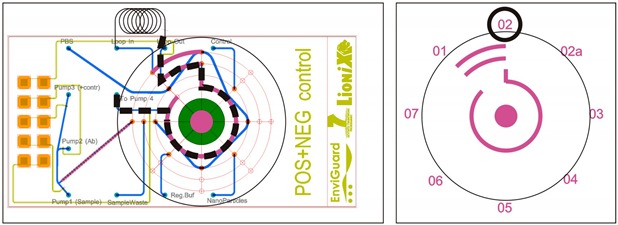
02a		
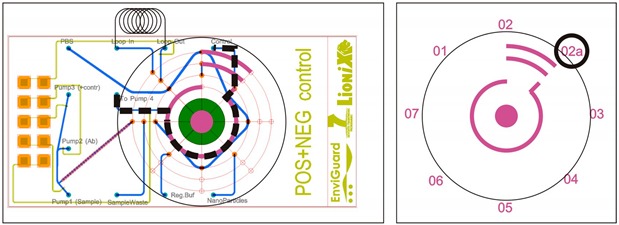
03		
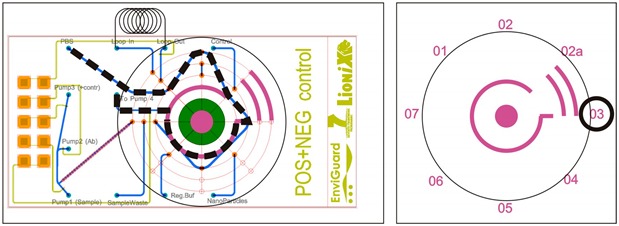
04		
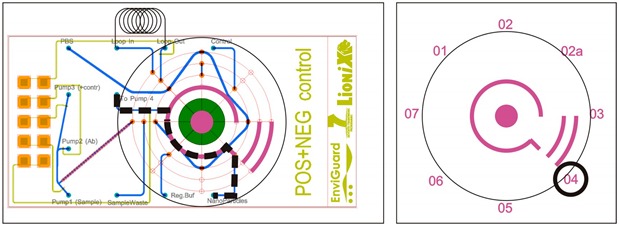
05		
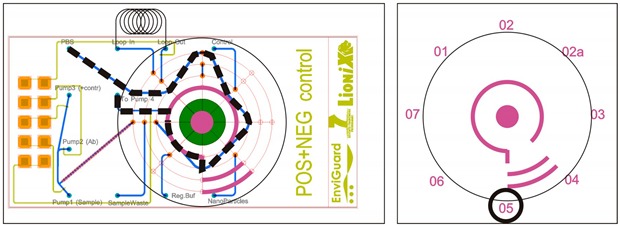
06		
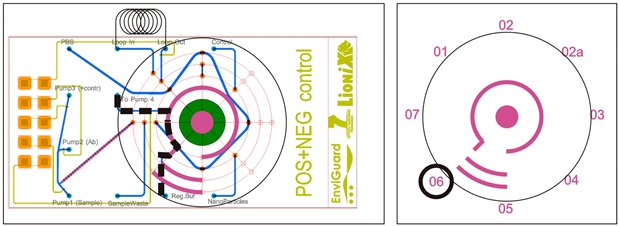
07		
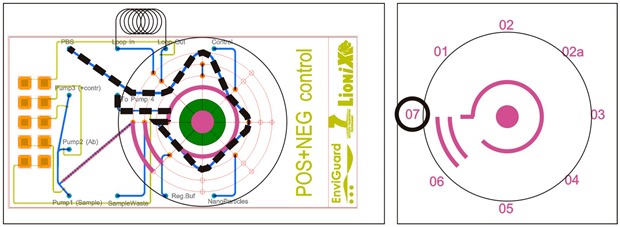

In order for the valve to operate leaktight, the rotor has to be compressed tightly onto the fluidic chip. An advanced chip-holder was developed (Figure A3), in which the fluidic valve-chip is kept in place, with compressed O-rings for the standard fluidic connections (10–32) towards the system.

A central axle is placed in the chip-holder, which aligns the rotor on the fluidic chip. A large gear is bolted to the rotor, such that a stepper motor can rotate the rotor by means of a secondary gear. Finally, the parts are pressed onto each other by a spring-loaded nut. The springs ensure that even with some temperature changes, the compression force between the parts stays in place. The realized valve assembly is shown in Figure A3.
